# In Vivo Observation of Trombiculosis with Fluorescence–Advanced Videodermatoscopy

**DOI:** 10.3201/eid2608.200077

**Published:** 2020-08

**Authors:** Alice Ramondetta, Simone Ribero, Andrea Peano, Pietro Quaglino, Paolo Broganelli

**Affiliations:** Università degli Studi di Torino, Turin, Italy

**Keywords:** mite, arthropod, fluorescence, videodermatoscopy, trombiculosis, vector-borne infections, parasites, Italy, Neotrombicula autumnalis, dermatitis

## Abstract

Trombiculosis is a skin infestation by larvae of mites of the Trombiculidae family. We used fluorescence–advanced videodermatoscopy to diagnose trombiculosis in a woman in Italy with targetoid patches. This method might be useful for identifying atypical manifestations of trombiculosis.

Trombiculosis is a skin infestation by mites of the Trombiculidae family. Adult mites live and reproduce on the surface of the soil, whereas the larvae feed on warm-blooded vertebrates, including humans. These mites are also known as harvest mites or chiggers (*1*–*6*). Although trombiculid mites are endemic to most of the world, certain soil conditions limit common habitats to grassy fields, forests, parks, gardens, and the moist areas along lakes and streams (*1*).

Only the larvae (0.15–0.3 mm long), which are 6-legged and orange or bright red, are responsible for chigger bites. Infestation usually occurs when larvae are particularly abundant, throughout late summer and autumn (*7*). Trombiculid larvae do not burrow into the host’s skin; instead, they use jaw-like structures to attach to hairless areas on the host, secrete digestive enzymes that liquify host epidermal cells, and feed on broken-down tissue and digested cutaneous cells for 2–10 days (*1*). The host response is mainly caused by sensitization to the injected saliva. Its intensity might vary from a slightly irritable erythema to papules or vesicles with intense itch (*3*).

Trombiculid mites are also vectors of infectious agents. For example, *Leptotrombidium* mites transmit scrub typhus in the Far East (*3*). We report a case of trombiculosis in a woman in Turin, Italy.

## Case Report

**Figure 1:**
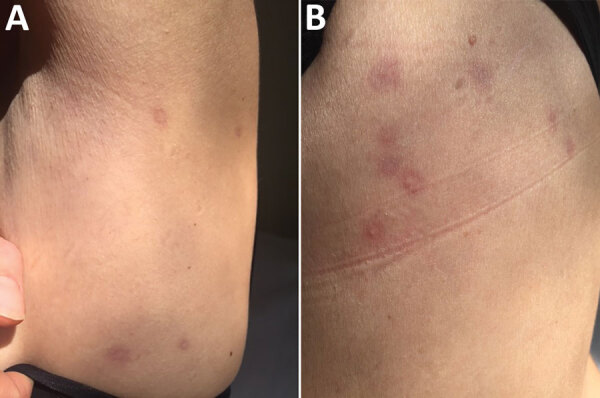
Roundish, targetoid patches, with peripheral erythematous halo and central lightening located on left abdomen (A) and on the back (B) of a 48-year-old woman with trombiculosis, Italy, April 2019.

In April 2019, a 48-year-old woman sought care at the Dermatology Clinic University Hospital of Turin, Turin, Italy, for onset of small roundish, targetoid patches, with peripheral erythematous halo and central lightening, on her trunk (Figure 1). The patient was otherwise in good health. A few days before the patches developed, she had gone on an excursion in the countryside near Turin.

We used fluorescence–advanced videodermatoscopy (FAV), an optical electronic system using a monochromatic light‐emitting source with an λ of 405 nm (±5 nm) and a field of view of 340 μm, to examine the patient. FAV uses the ability of endogenous molecules to absorb specific wavelengths and emit fluorescence. The examination is conducted in vivo; the optical device is directly applied to the skin. The images are visualized in real time by using grayscale to indicate the levels of light absorption (i.e., black indicates no fluorescence, and white indicates highest fluorescence) (*8*).

**Figure 2:**
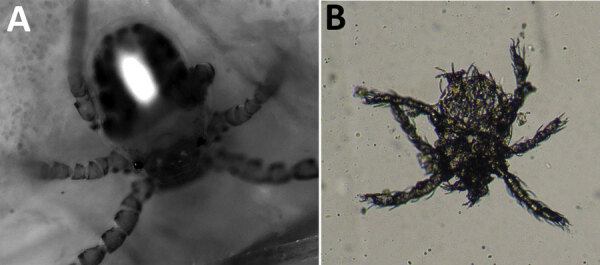
Images of trombiculid mites from infestation of a 48-year-old woman, Italy, April 2019. A) Fluorescence–advanced videodermatoscopy image shows oval body with 3 pairs of legs. Original magnification ×500. B) Optical microscope examination of 1 larva detected from a superficial skin scraping. Original magnification ×100.

By placing the probe on one of the patient’s skin patches, we visualized an oval-shaped mite with 3 pairs of legs (Figure 2, panel A). We identified this image as the larval stage of a trombiculid mite. We also detected 1 larva on direct examination from a superficial skin scraping (Figure 2, panel B). The patient was treated with topical ivermectin for 7 days, resulting in complete regression of the lesions.

## Conclusions

FAV facilitated a real-time diagnosis of trombiculosis in this case. Without this technology, the diagnosis of trombiculosis might have been complicated by the atypical presentation (trombiculosis usually manifests with erythematous homogeneous macules) and seasonality of the case (in Italy, trombiculosis is more prevalent in autumn [*7*]). To our knowledge, the diagnosis of trombiculosis using videodermatoscopic findings has been reported in only 1 other case (*6*).

We suspect the agent responsible for this case was the *Neotrombicula autumnalis* mite, the most frequent causative agent of trombiculosis in Europe (*2*–*5*). However, because we could not collect larvae to perform morphologic or molecular studies, we could not definitively identify the mite. At least 4 other species of trombiculid mites might cause trombiculosis in Europe, namely *Kepkatrombicula desaleri*, *Blankaartia acuscutellaris*, *Trombicula toldti*, and *N. inopinata* (*9*). In addition, the number of cases caused by species other than *N. autumnalis* might be underestimated because, in most reports, the identification does not appear to have been attributed with sufficient taxonomic criteria (*9*).
